# Magnetic Resonance Imaging Features and Functional Score in Patients Requiring Total Knee Arthroplasty

**DOI:** 10.7759/cureus.68595

**Published:** 2024-09-04

**Authors:** Sergiu Iordache, Adrian Cursaru, Andreea Marinescu, Bogdan Cretu, Mihnea Popa, Mihai Aurel Costache, Bogdan Serban, Catalin Cirstoiu

**Affiliations:** 1 Orthopaedics and Traumatology, University Emergency Hospital, Bucharest, ROU; 2 Radiology and Imaging, University Emergency Hospital, Bucharest, ROU

**Keywords:** modified knee society clinical score (kss), mri imaging, articular cartilage, knee arthroplasty, functional score, osteoarthrosis

## Abstract

Introduction

Knee osteoarthritis (KOA) is a progressive degenerative disease, with an increasing prevalence among the population. The degenerative changes in KOA affect the cartilage, menisci, synovial tissue, and subchondral bone. The treatment for patients in advanced stages of the disease is total knee arthroplasty (TKA).

The purpose of this descriptive study is to identify the MRI features in the case of patients with KOA who did not obtain an improvement in symptoms and joint function after the non-surgical treatments and who applied for surgical treatment, i.e. TKA. Also, we aimed to identify the correlations between the MRI changes and the functional score of the patients, as well as the inter-variable correlations.

Materials and methods

The study was conducted in the Department of Orthopedics and Traumatology at the University Emergency Hospital of Bucharest between January 1, 2023, and January 31, 2024. It included 50 patients who required TKA. This study is a prospective, observational, and descriptive analysis focusing on patients scheduled for TKA.

Results

The patients in the study group who required TKA had a Knee Society Score (KSS) ranging from 35 to 70 and a KSS function score between 24 and 60. Among them, 98% had tricompartmental lesions of the articular cartilage, and 100% presented with osteophytes, even when they were not identifiable radiologically. Additionally, 58% of the patients had changes in the infrapatellar fat pad, 66% presented with joint effusion without any traumatic history, and 86% of the patients had partial or complete lesions of the anterior cruciate ligament.

Conclusion

The MRI pattern of the patient who requires TKA consists of the presence of articular cartilage lesions in more than two compartments with exposure of the subchondral bone and diameter greater than 1 cm, meniscus lesions (>grade 2), meniscus extrusion (>grade 1), the presence of bone marrow lesions (BMLs) in the medial or lateral compartment of the femur or tibia, hyperintense signal of the infrapatellar fat pad, anterior cruciate ligament (ACL) lesions (>grade 2), and the presence of osteophytes together with the presence of effusion in the suprapatellar bursa.

BMLs and changes in the infrapatellar fat pad may lead to the opening of new research perspectives explaining the complex changes in KOA in relation to the inflammatory process and gene expression.

## Introduction

Knee osteoarthritis (KOA) is a degenerative disease and the number of people affected by this condition is increasing. In a study conducted from the data of the National Health Interview survey, it emerged that the prevalence of KOA in the USA was six million people aged between 45-65 years and almost two million people under 45 years old [1,2].

Degenerative changes in knee osteoarthritis involve cartilage, menisci, synovial tissue, and subchondral bone. The most common symptoms that appear are pain, edema and gait difficulties.

Osteoarthritis usually develops progressively over the years, but symptoms can remain stable for long periods. Almost half of the patients with radiological imaging characteristics of KOA have no symptoms or vice versa [1].

The main treatment for patients in the final stage of the disease is total knee arthroplasty (TKA). The knee prosthesis has been used for more than 40-50 years, and its effectiveness has been validated in large studies. The purpose of total knee arthroplasty is to reduce pain and restore joint function [3].

Currently, the gold standard for the assessment of arthritic changes is radiography. During the evolution of knee osteoarthritis, several stages can be distinguished: pre-clinical, pre-radiological, and final. The classification system proposed by Kellgren and Lawrence is a classic method for diagnosing and classifying knee osteoarthritis on radiographs and includes joint space narrowing, subchondral bone sclerosis, and presence of osteophytes [4,5]. It was proposed in 1957 and accepted by the World Health Organization (WHO) in 1961 for research purposes.

The classification includes five grades as follows: grade 0, absence of X-ray changes of osteoarthritis; grade 1, doubtful joint space narrowing and possible osteophytic lipping; grade 2, osteophytes and possible joint space narrowing; grade 3, moderate multiple osteophytes, narrowing of joint space, some sclerosis and possible deformity of bone ends; and grade 4, large osteophytes, marked narrowing of joint space, severe sclerosis and definite deformity of bone ends [6-10].

Unfortunately, this has certain limitations, namely, it can only evaluate calcified tissues and joint space narrowing [10,11]. A much more sensitive imaging method than the conventional one in the evaluation of arthritic changes is magnetic resonance imaging (MRI). This specialized imaging investigation, which can evaluate the structural changes of the entire joint in a 3D way, occurring at the level of the joint like cartilage lesions, synovial changes, meniscal tears, lesions occurring at the subchondral bone level in an early stage, pre-radiological [12-14]. It should be mentioned that synovial inflammation can occur both in the early stages of the disease and in the advanced stages, being responsible for local edema and pain [15].

MRI plays an important role in the assessment of knee osteoarthritis progression not seen on radiographs [16]. MRI evaluation of cartilage damage, meniscus tears, and synovitis can be sensitive imaging biomarkers regarding the progression of osteoarthritis over time and the subsequent total knee arthroplasty [15].

Large studies have evidenced that the loss of tibial cartilage is an independent predictor of replacing the joint with a knee prosthesis [17-19]. Another study showed that patients with greater cartilage loss in the central and medial tibiofemoral compartment compared to the control group were more likely to undergo total knee arthroplasty [18-21].

More and more patients have knee pain but without radiological osteoarthritis changes. In clinical practice, it is not clear how to manage these patients and whether the additional MRI imaging would be of clinical value, whether it could predict the degree of probability in the event of a knee arthroplasty in the future. Such data can only be collected in prospective studies because people without KOA are not usually included in studies [8,22].

Therefore, the aim of this study is to discover structural anomalies that can be observed on MRI but not conventionally (radiograph) and that can be used as predictors in the evolution of KOA and the need for total knee arthroplasty. The most important question is whether MRI can be useful in the early diagnosis of patients who require TKA [23-25].

## Materials and methods

The study was conducted in the Department of Orthopedics and Traumatology of the University Emergency Hospital of Bucharest between January 1, 2023 to January 31, 2024 and included 50 patients who required total knee arthroplasty. The study carried out is a prospective, observational and descriptive type on patients who were proposed for total knee arthroplasty. For every patient in addition to conventional imaging (radiographs), the preoperative functional score was also calculated. Each patient included in the study underwent a 3 Tesla MRI of the knee to identify associated injuries and also identification of MRI imaging pattern in the case of patients who need total knee arthroplasty. The inclusion criteria for the study were patients with knee pain, with radiological changes of KOA, aged over 40 years, to whom all non-surgical treatments were applied, without improvement at the time of presentation in our department. The non-surgical treatments used by the patients were represented by the administration of non-steroidal anti-inflammatory drugs, physiotherapy, the recommendation of weight loss (unsuccessful in all cases), the administration of intra-articular steroids and intra-articular viscoelastic supplementation (Table 1).

**Table 1:** Non-surgical treatment methods and their duration applied to patients together with BMI values ​​and functional score NSAID-Non-Steroidal Anti-Inflammatory drugs BMI-Body Mass Index KSS-Knee Society Score

	Age	Sex	NSAIDs (Yes/No, Duration-months)	Physiotherapy (Yes/No, Duration-months)	Weight loss In the last year (Yes/No)	Intra-articular steroids (Yes/No)	Intra-articular viscoelastic supplementation (Yes/No)	BMI N value=18.5-24.9)	KSS Knee score	KSS Function score
1	67	F	Yes/ 12	Yes/ 6	No	No	Yes	34	60	60
2	68	F	Yes/8	Yes/3	No	Yes	No	31	50	42
3	81	F	Yes/12	Yes/6	No	Yes	Yes	30.2	50	50
4	64	M	Yes/9	Yes/6	No	No	Yes	34.2	60	50
5	51	F	Yes/12	Yes/6	No	Yes	Yes	36.7	60	50
6	62	F	Yes/8	Yes/9	No	No	Yes	35.8	70	60
7	67	F	Yes/6	Yes/6	No	Yes	Yes	33.6	70	60
8	67	F	Yes/12	Yes/12	No	No	Yes	34.9	35	24
9	54	F	Yes/6	Yes/6	No	No	Yes	33.8	70	60
10	68	F	Yes/9	Yes/6	No	Yes	No	26.4	70	60
11	65	M	Yes/24	Yes/12	No	No	Yes	30.9	60	50
12	68	F	Yes/28	Yes/12	No	Yes	Yes	28.3	35	35
13	74	M	Yes/10	Yes/6	No	No	Yes	22.5	60	50
14	69	F	Yes/8	Yes/8	No	Yes	Yes	33.8	60	60
15	65	F	Yes/12	Yes/8	No	No	Yes	27	60	50
16	56	F	Yes/10	Yes/6	No	No	Yes	34.3	70	60
17	64	F	Yes/16	Yes/8	No	Yes	Yes	30.9	60	50
18	67	F	Yes/12	Yes/6	No	Yes	Yes	33.2	60	60
19	55	M	Yes/14	Yes/6	No	No	Yes	28.1	60	50
20	77	F	Yes/12	Yes/8	No	Yes	Yes	30.1	35	24
21	69	M	Yes/16	Yes/9	No	No	Yes	31	60	50
22	75	F	Yes/18	Yes/9	No	Yes	Yes	29.2	60	35
23	62	F	Yes/14	Yes/12	No	No	Yes	34.5	60	35
24	74	F	Yes/26	Yes/10	No	Yes	Yes	32	35	24
25	59	M	Yes/14	Yes/8	No	No	Yes	26.1	70	60
26	65	M	Yes/12	Yes/6	No	Yes	Yes	32	60	50
27	62	F	Yes/28	Yes/10	No	Yes	Yes	31.4	35	24
28	72	F	Yes/10	Yes/6	No	No	Yes	30.2	60	50
29	73	F	Yes/15	Yes/6	No	No	Yes	29.1	60	42
30	71	M	Yes/24	Yes/10	No	Yes	Yes	29.2	35	35
31	44	F	Yes/26	Yes/8	No	Yes	Yes	31.2	35	35
32	76	F	Yes/16	Yes/6	No	Yes	Yes	31.4	40	24
33	76	F	Yes/12	Yes/9	No	Yes	Yes	30	60	50
34	55	M	Yes/10	Yes/9	No	Yes	Yes	29.2	70	50
35	58	F	Yes/12	Yes/6	No	Yes	Yes	28.1	60	42
36	71	M	Yes/14	Yes/6	No	No	Yes	30.1	60	50
37	75	F	Yes/16	Yes/4	No	Yes	Yes	33.2	35	35
38	56	F	Yes/22	Yes/8	No	Yes	Yes	29.8	60	50
39	62	M	Yes/12	Yes/6	No	Yes	Yes	27.2	60	50
40	72	F	Yes/18	Yes/9	No	Yes	Yes	30.2	35	35
41	45	F	Yes/12	Yes/6	No	No	Yes	31	70	50
42	69	F	Yes/14	Yes/8	No	No	Yes	29.2	60	50
43	64	F	Yes/16	Yes/6	No	Yes	Yes	27.5	70	42
44	69	F	Yes/18	Yes/9	No	Yes	Yes	29.7	60	50
45	59	F	Yes/12	Yes/6	No	No	Yes	30.4	70	50
46	63	F	Yes/14	Yes/6	No	Yes	Yes	31	70	50
47	65	F	Yes/9	Yes/6	No	No	Yes	29.8	70	60
48	74	F	Yes/18	Yes/9	No	Yes	Yes	33.5	50	24
49	59	M	Yes/18	Yes/9	No	Yes	Yes	33	50	42
50	53	F	Yes/12	Yes/6	No	Yes	Yes	29.8	60	50

The exclusion criteria consisted of septic arthritis of the knee, a recent traumatic episode that coincided with the onset of pain, the presence of autoimmune diseases in the algic phase or any other systemic disease with joint involvement, the refusal to perform an MRI for personal reasons and the lack of any medical records related to previous KOA treatments. Study approval was obtained from the local ethics committee at the University Emergency Hospital of Bucharest and informed consent was obtained from all participants (40545/2023).

Statistical analysis was performed using SPSS Statistics 21 (IBM Corp., Armonk, NY, USA) and MedCalc® Statistical Software version 22.026 (Ostend, Belgium). Data were collected using Excel 6.2.14(v16.0) (Microsoft, Redmond, WA, USA). The collected data were age, gender, the Oxford Knee Society Score calculated at presentation and the radiological stage established using the Kellgren and Lawrence classification together with the MRI changes.

Therefore, the purpose of this descriptive study is to identify the MRI features in the case of patients with KOA who did not obtain an improvement in symptoms and joint function after the non-surgical treatments listed in Table 1 and who applied for surgical treatment, i.e. total knee arthroplasty. Also, the identification of the correlations between the MRI changes and the functional score of the patients, as well as the inter-variable correlations. The purpose of the MRI examination is to open new research perspectives and to help, based on existing observations, to identify future patients who will require total knee arthroplasty. The usefulness of the MRI examination is addressed especially to patients who do not have significant radiological changes but present significant pain that does not improve with non-surgical treatment and in which no other cause of pain can be identified.

The MRI imaging examination was performed by a single radiologist superspecialized in musculoskeletal disorders to avoid interobserver variations. Also, the examination was done according to a standardized protocol. As part of the MRI analysis protocol, we analyzed the condition of the articular cartilage in the femorotibial and femoropatellar compartment, mensical lesions, presence or absence of effusion, bone marrow lesions (BMLs), changes in the infrapatellar fat, associated lesions of the anterior cruciate ligament, bone deformities with bone wear specific to the knee in varus or valgus, presence of osteophytes or synovial fold. Articular cartilage lesions refer to full-thickness cartilage loss (grade 3) in any of the compartments and with a diameter greater than 1 cm. Cartilage is considered normal if it has a uniform thickness. We defined meniscal pathology as the presence of a meniscal extrusion (>grade 1) or the presence of a mensical tear (>grade 2) [26]. Knee effusion represents the presence of synovial fluid in the joint space of the knee. The effusion volume was evaluated by analyzing the suprapatellar bursa according to the intraarticular fluid-equivalent signal on an MRI, section-by-section basis and was recorded as present or absent. BMLs were defined as areas of increased signal located adjacent to the subcortical bone in the medial or lateral compartments of the femur and tibia. The infrapatellar fat pad was analyzed as signal intensity on the MRI sequences, taking into account the fact that the hypointense aspect is specific to fibrosis and the hyperintense aspect is specific to inflammation. Changes in the infrapatellar fat pad were noted as present or absent only in cases with a hyperintense signal, specific to inflammation. The anterior cruciate ligament was included in our study because it is the main affected ligament of the knee and its injury leads to the establishment and worsening of KOA. Anterior cruciate ligament injuries were noted as normal, partial and complete injury. Bone deformities were recorded as present or absent by assessing the weight-bearing portion of the knee compartments and areas with osteocondensation >10mm were defined as present. Osteocondensation is defined as the increase in bone density due to chronic bone wear. Marginal osteophytes were defined as present if more than two were detected in different areas. The synovial fold was recorded as present or absent regardless of the region where it is located.

Statistical tests used in the analysis

Chi-square test is used to check if two nominal/dichotomous variables are associated. The Phi correlation coefficient is used to identify associations between two dichotomous variables and takes values ​​in the range of -1 to 1. The direction of the association is given by the sign. Cramer's V correlation coefficient measures the strength of association between variables and takes values ​​between 0 and +1. A p-value <0.05 was considered statistically significant. Normality of distributions was established using the Kolmogorov-Smirnov and Shapiro-Wilk tests.

## Results

Fifty eligible patients were included with an average age of 65.12 years, of which 12 were men and 38 were women (Table 2). The patients had a BMI between 22.50 and 36.70, with a mean value of 30.79 (standard deviation 2.73; CI: [30.01, 31.57]) and a median value of 30.65. No significant gender differences were noted in total knee arthroplasty patients' BMI, although women had a slightly higher BMI than men (mean BMI women 31.21 vs. men 29.45).

**Table 2:** Patient Group Description *Age is quantified in years

Demographic data	Total (N=50)
Average age	65.12
Minimum age	44
Maximum age	81
95% confidence interval for mean	62.79-67.45
Median	66
Standard Deviation	8.19
Gender distribution	12 men
	38 women
Average age men	64.08
Average age women	65.45

Preoperative functional score of patients

Total knee arthroplasty patients had KSS scores ranging from 35 to 70, with a mean value of 56.70 (standard deviation 12.02; CI: [53.28, 60.12]) and a median value of 60.

Total knee arthroplasty patients had KSS function scores ranging from 24 to 60, with a mean value of 45.98 (standard deviation 11.19; CI: [42.80, 49.16]) and a median value of 50.

MRI changes

In the studied group, tricompartmental lesions predominate, these being found in 49 (98%) patients, while lesions in one compartment are rarely found, in only one (2%) patient. There were no cases with bicompartmental lesions.

Most of the patients presented bone marrow lesions, these being present in 47 (94%) cases, while their absence was found in only three (6%) patients.

Almost all patients presented bone deformities, these being present in 47 (94%) cases, while their absence was found in only three (6%) patients.

Although the MRI is not a gold standard investigation for bone imaging, the presence of osteophytes was 100%. Osteophytes were also identified by simple X-rays performed when the patient was admitted to the orthopedic department.

Mensical lesions were present in most of the patients, thus being present in 48 (96%) cases, while their absence was found in only two (4%) patients.

Twenty-nine (58%) patients had inflammatory changes regarding the infrapatellar fat pad presenting a hyperintense signal in the region. Twenty-one (42%) presented a hypointense signal on the MRI sections or did not present any changes in the infrapatellar fat pad.

Most of the patients in the studied group presented effusion at the time of admission, more precisely 33 (66%), without having a recent trauma or other systemic disease that generates effusion.

**Figure 1:**
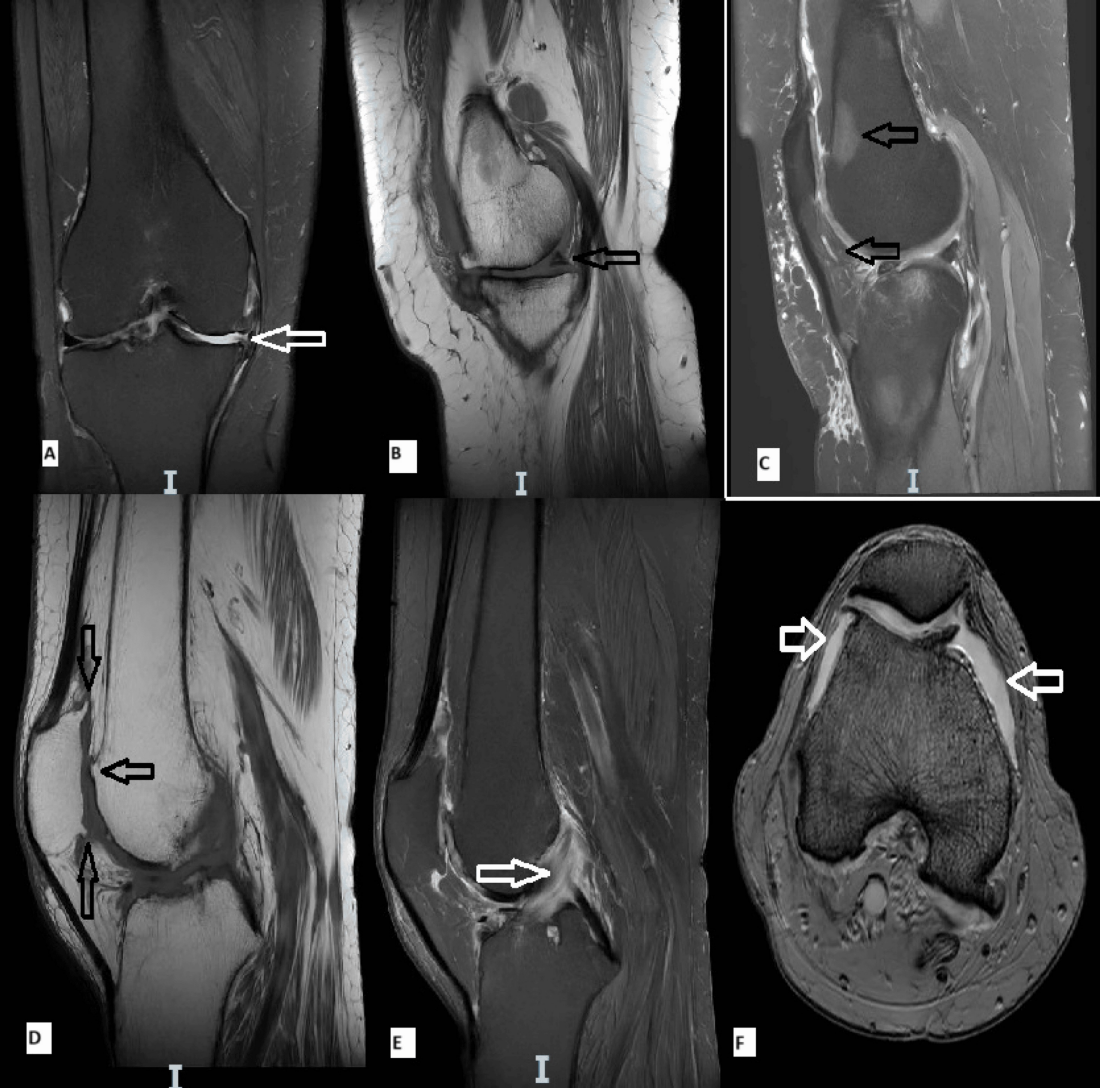
3T MRI images of the patients A-Important reduction of the joint space and loss of the articular cartilage-T2/coronal view B-Grade 3 tear of the posterior horn of internal meniscus associating the anterior subluxation of the anterior horn-T1/sagittal view C-Upper arrow shows localized bone edema at the femoral-patellar level and significant cartilage loss; Bottom arrow showing infiltrative hypersignal of Hoffa fat pad-T2/sagittal view D-Osteophytes-T1/sagittal view E-Partial anterior cruciate ligament injury-T2/sagittal view F-Effusion-T2/Axial view

Patients frequently presented ligament injuries, with their distribution being as follows: 23 (46%) had complete anterior cruciate ligament (ACL) injury, 20 (40%) had partial ACL injury and seven (14%) had no changes (Table 3). No patient presented a recent trauma, so the identified lesions are either chronic injuries or degenerative changes in the evolution of KOA (Figure 1).

**Table 3:** Distribution of cases according to MRI changes (Data represented as % and N) BMLs-Bone marrow lesions, ACL-anterior cruciate ligament

MRI targets	Features	N (%)
Meniscal tears	Absent	2(4%)
	Present	48(96%)
Effusion	Absent	17(34%)
	Present	33(66%)
BMLs	Absent	3(6%)
	Present	47(94%)
Bone deformities	Absent	3(6%)
	Present	47(94%)
Infrapatellar Fat Pad	Normal	21(42%)
	Modified	29(58%)
Ligament Tears (ACL)	No tear	7(14%)
	Partial tear	20(40%)
	Complete tear	23(46%)

It was observed that 29 (61.70%) patients with bone edema had altered infrapatellar fat pad. Infrapatellar fat pad showed no changes in patients without bone edema (p<0.05).

The application of a Chi-square test indicated that between the presence of bone edema and the change in infrapatellar fat pad there is a statistically significant association presenting the following result χ2(1, N=50)=4.407, p=0.036, and Cramer's V and Phi coefficients (φ=0.297, p=0.036) reveal that between the two variables is a direct association, but weak in intensity. This statistical test indicates that the presence of bone edema is associated with altered infrapatellar fat (Table 4).

**Table 4:** Distribution of cases according to the associations of MRI changes (Data represented as N and %) BMLs-Bone marrow lesions, ACL-anterior cruciate ligament

MRI features	Associated MRI features	Present N (%)
BMLs	Infrapatellar fat	29 (61.7%)
	Effusion	30 (63.83%)
No ACL tear	Bone deformities	5 (71.43%)
Partial ACL tear	Bone deformities	20 (100%)
Complete ACL tear	Bone deformities	22 (95.65%)

It was noted that patients with anterior cruciate ligament tears (partial or complete) had significantly more frequent bone deformities. Thus, patients with no ligament injuries presented bone deformities in five (71.43%) cases while all patients with partial ACL injury presented bone deformities (20; 100%). Regarding the patients with complete ACL tears, 22 (95.65%) presented bone deformities. It was observed that patients with partial or complete lesions of the anterior cruciate ligament presented more frequent bone deformities compared to patients who had an intact anterior cruciate ligament. This can be explained by the knee instability generated by ACL laxity.

The application of a Chi-square test indicated that between the presence of ligament injuries and the presence of bone deformities there is a statistically significant association, recording the following result χ2(2, N =50)=7.711, p=0.021. Cramer's V coefficient and Phi coefficient (φ=0.393, p=0.021) reveal that the association between the two variables is a direct one and moderate in intensity. This statistical test shows that patients with ligament injuries have a moderate tendency to show bone deformities (Table 4).

Other aspects related to MRI changes

Although they did not mark any significant associations between the parameters, the following aspects related to the MRI changes of patients with total knee arthroplasty were noted. Only patients with ligament injuries (partial or complete) presented synovial fold, thus this was not found in any patient without ligament injuries (N=10 (50%), N=10 (43.48%), N=0 (0%), (p>0.05)).

Patients with BMLs presented less frequently effusion than those without bone edema (N=30 (63.83%) vs. N=3 (100%), (p>0.05)). Only patients with meniscal lesions presented complete ACL lesions (N=23 (47.92%) vs. N=0 (0%), (p>0.05)).

There were no cases without bone edema presenting synovial fold (N=0 (0%) vs. N=20 (42.55%), (p>0.05)). The synovial fold was found only in cases that presented bone deformities (N=20 (42.55%) vs. N=0 (0%), (p>0.05)). Patients with effusion had a synovial fold more frequently than those without effusion (N=15 (45.45%) vs. N=5 (29.41%), (p>0.05)).

Patients with meniscal tears had a lower KSS score than those without lesions (mean KSS score 56.35 vs. 65). They also had a lower functional KSS score than patients without meniscal lesions (mean functional KSS score 45.60 vs. 55), (p>0.05) (Table 5).

**Table 5:** The main MRI changes that significantly influenced the KSS value (Data presented as mean value) KSS-Knee Society Score, BMLs-Bone marrow lesions

MRI features	Present/Absent	KSS (mean value)	KSS function (mean value)
Mensical tears	Present	56.35	45.60
	Absent	65	55
BMLs	Present	45	39.67
	Absent	57.45	46.38

Patients with BMLs had a slightly lower KSS score compared to patients with bone edema (45 vs. 57.45). They also had a slightly lower KSS function score than patients without bone edema (39.67 vs 46.38) (p>0.05) (Table 5).

## Discussion

The MRI represents a high-performance imaging investigation useful in the assessment of soft tissue injuries such as the menisci, infrapatellar fat pad, synovial tissue, ligaments, muscle structures, the presence of effusion, identification of bone edema, but also the bone structure. Thus, knee osteoarthritis is no longer considered an exclusive bone and cartilage disease, the damage being a complex one involving all knee structures. Pain and joint function are influenced not only by the degree of osteo-cartilaginous damage but also by the associated lesions of the soft tissues.

Quantifying the thickness of the articular cartilage and identifying its lesions using MRI is a risk factor for progression to total knee arthroplasty. Thus, within the studied group, tricompartmental lesions of the articular cartilage were identified in 49 (98%) of the patients who required total knee arthroplasty, in accordance with the results of Roemer et al. who identified the lesions of the articular cartilage located at the level of two or three compartments as a risk factor for TKA [22].

Lo et al. recognize bone edema as an imaging marker of joint pain, and its presence can lead to a decrease in functional scores, in many situations without direct association related to the degree of osteo-cartilaginous damage. Forty-seven (94%) of the patients included in the study presented BMLs, which is in agreement with the results of Lo et al. [23]. Also, several longitudinal studies found that the severity of BMLs was positively associated with cartilage defect, cartilage volume loss, joint space narrowing and joint replacement [24]. 

Infrapatellar fat pad has recently been recognized as having a key role in the evolution and progression of KOA. In MRI, the infrapatellar fat pad structure appears hypointense with lower signal foci throughout the tissue. Twenty-nine (58%) of the patients included in the study who required TKA showed MRI changes of the infrapatellar fat pad, which explains the evolution and severity of symptoms of the patients. Wu et al. identified a direct association between the serum level of ghrelin and the MRI changes of the infrapatellar fat pad, as well as the associations with IL-17 and resistin, explaining the symptoms and progression in patients with KOA [25].

The menisci have a crucial role in shock absorption and can be affected during the evolution of KOA, from radial, longitudinal injuries, their combinations causing complex injuries or meniscus extrusion. Most injuries in patients with KOA do not appear as a result of a major trauma but are a consequence of meniscal degeneration. In the analyzed group of patients, 48 (96%) presented meniscal lesions, and in the case of patients with meniscus lesions, a lower KSS score was recorded compared to the other patients, as presented by Driban et al. on a larger group of patients [27]. The excess synovial tissue, its thickness and its extension at the level of the knee joint is in many situations the consequence of the evolution and progression of KOA, but it can also reveal the presence of an autoimmune inflammatory disease.

Within the analyzed study group the presence of effusion together with articular cartilage lesions, meniscal lesions, the presence of osteophytes and BMLs are significantly associated with the development of radiological signs of KOA [22-24,27]. The detection of a synovial fold can help, depending on its location, in the differential diagnosis of knee pain.

The limitations of the study are determined by the lack of a homogeneous sample of patients (women>men), the lack of data at the time of onset of symptoms and the relatively small number of patients to show statistical significance.

## Conclusions

The results of the study are in accordance with the data from the literature and identify direct correlations between the MRI changes and the functional scores calculated preoperatively. Thus, the patients who required total knee arthroplasty in the studied group presented a KSS score between 35-70, KSS function score between 24-60 and 49 (98%) patients had tricompartmental lesions of the cartilage.

Thus, developing an MRI pattern of the patient who requires total knee arthroplasty, we can list the presence of articular cartilage lesions in more than two compartments with exposure of the subchondral bone and diameter greater than 1 cm, meniscus lesions (>grade 2), meniscus extrusion (>grade 1), the presence of BMLs in the medial or lateral compartment of the femur or tibia, hyperintense signal of the infrapatellar fat pad, ACL lesions (>grade 2), the presence of osteophytes together with the presence of effusion in the suprapatellar bursa.

BMLs and changes in infrapatellar fat pad may lead to the opening of new research perspectives explaining the complex changes in KOA related to the inflammatory process and gene expression. The positive correlation between ligament injuries and bone deformations can be explained by the biomechanical damage of the joint.
